# Targeted therapeutic strategies as alternative and sustainable treatment options for obesity-induced steatohepatitis

**DOI:** 10.1007/s11154-025-09980-7

**Published:** 2025-06-25

**Authors:** Thendo I. Mabuda, Nicole R. S. Sibuyi, Adewale O. Fadaka, Mervin Meyer, Abram M. Madiehe, Kwazikwakhe B. Gabuza

**Affiliations:** 1 Nanobiotechnology group, Department of Biotechnology, University of the Western Cape, Bellville, 7535 South Africa; 2 Department of Science, Technology and Innovation/Technology Innovation Agency Nanotechnology Platform, Department of Biotechnology, University of the Western Cape, Bellville, 7535 South Africa; 3 Biomedical Research and Innovation Platform, South African Medical Research Council (SAMRC), Tygerberg, 7500 South Africa; 4 Health Platform Diagnostic Unit, Advanced Materials Division, Mintek, Randburg, 2194 South Africa; 5 Centre for Cardio-Metabolic Research in Africa, Department of Physiological Sciences, Stellenbosch University, Cape Town, South Africa

**Keywords:** Obesity, Targeted therapy, Metabolic syndrome, Metabolic dysfunction-associated steatotic liver disease, Metabolic dysfunction-associated steatohepatitis

## Abstract

The development of metabolic dysfunction-associated steatotic liver disease (MASLD), and its progression to metabolic dysfunction-associated steatohepatitis (MASH), is triggered and aggravated by obesity and related metabolic diseases. Hence, therapeutic strategies for obesity and diabetes are employed in the clinical management of MASLD and MASH. However, most of the antiobesity and antidiabetic drugs have been withdrawn from the market due to a lack of sustainable drug effects and undesirable side effects. Prior to the approval of resmetirom by the United States Food and Drug Administration in 2024, there were no specific treatments for MASLD and MASH. Therefore, this stresses the importance of developing improved and effective disease-specific treatment strategies to combat MASLD and MASH. MASLD and MASH are characterized by hepatocyte damage, chronic low-grade systemic inflammation, and abnormalities in lipid metabolism. Inflammation and oxidative stress are key mediators of high-fat diet-induced steatohepatitis and obesity. Identifying biomarkers involved in these processes could aid in the development of targeted treatment for MASLD and MASH. In this review, targeted treatment strategies for obesity are discussed in relation to the possibility of the same being used in obesity-related steatotic liver diseases (SLDs).

## Introduction

MASH is characterized by excessive lipogenesis and severe inflammation of the liver tissue. Without treatment, MASH may progress to more severe stages of the disease, such as cirrhosis and ultimately hepatic carcinoma [1, 2]. In obese individuals, MASH may be triggered by several factors, such as decreased fatty acid β-oxidation in the mitochondria, increased fatty acid delivery to the liver, *de novo* lipogenesis, and inadequate export or integration of lipids within hepatic cells [3, 4].

Current efforts for the management of MASH rely on strategies used for obesity and diabetes, which involve pharmacotherapy and lifestyle modifications. These strategies mainly focus on reducing body weight and improving metabolic activities [5]. Obesity treatments are currently focused on multi-target drugs, including next-generation incretin-based therapies and gene therapy approaches, as well as gut microbiome modulation [6]. However, lifestyle modification alone or in combination with pharmacotherapy, is often insufficient to achieve these effects, let alone to sustain body weight lost [7]. Moreover, most of these drugs have been withdrawn from the market due to their harmful side effects [8, 9]. This has exacerbated the obesity epidemic and MASH over the years, stressing the importance of developing safe and effective treatment strategies. Although, the approval of resmetirom, an oral, thyroid hormone receptor-β agonist, marks a significant progress in the treatment of MASLD [10], it is imperative to acknowledge that other potential alternative therapies are still needed.

Advancements in targeted therapies have gained recognition in the management and treatment of chronic diseases [11–13]. Targeting biomarkers that are exclusively expressed in a disease of interest have proven to enhance bioavailability and limit premature drug clearance [14]. Therefore, employing treatments that target molecules linked to SLD development and progression may offer a more specific and effective therapeutic strategy for MASLD and MASH. Metabolic diseases, inclusive of MASLD, are preceded by inflammation mediated by immune cells, characterized by the migration of neutrophils and macrophages [15, 16]. This process is regulated by multifunctional cell membrane proteins, such as prohibitin (PHB), vascular endothelial growth factor (VEGF), CD31, CD44, CD68 and hepatocyte growth factor (HGF) [17–19]. Studies targeting disease-specific biomarkers have shown improvement in drug efficacy, by enhancing the selectivity and biodistribution of therapeutic agents in diseased cells [20, 21]. Similarly, molecular mechanisms that modulate processes involved in the development of MASLD and MASH have been investigated [4], and biomarkers involved in these processes could be used as targets for therapeutic interventions.

## The prevalence of MASLD

MASLD is considered the hepatic manifestation of the metabolic syndrome and a serious health threat, as its overall burden has escalated due to the uncontrolled increase in metabolic diseases [22]. The prevalence of MASLD in Africa is estimated to be 13.5% of the adult population [23]. This is viewed as an underestimate due to the lack of updated reports, coupled with the increase in obesity [23, 24]. Globally, 39% of the population is overweight, 13% is obese, 6.28% suffer from type 2 diabetes, and 31.1% are diagnosed with hypertension [25–27]. The prevalence of MASLD among patients with type 2 diabetes varies by country. For example, Ethiopia had the highest rate at 73%, followed by Nigeria with 69%, and 50% in Sudan [28]. One study in South Africa estimated a prevalence of 48%. The study reported that from a cohort of 233 patients, mixed-race individuals (69%) were more prone to developing SLD and mild fibrosis when compared to Caucasian (25%) and black (5%) individuals [29].

The global prevalence of MASLD has significantly increased over the years, from an estimated 25.5% in 2005 to an estimated 37.8% reported in 2016 and beyond. These statistics represent 39.9% of adult males and 25.6% of adult females [30]. MASLD remains the most common etiology of chronic liver disease at 59% when compared to hepatitis B (29%) > hepatitis C (9%) > alcoholic liver disease (2%), and other liver diseases (1%) [31, 32]. It is, therefore, predicted to become the main cause of cirrhosis and hepatic carcinoma, due to its pathogenesis [31, 32].

## MASLD pathogenesis

**Fig. 1:**
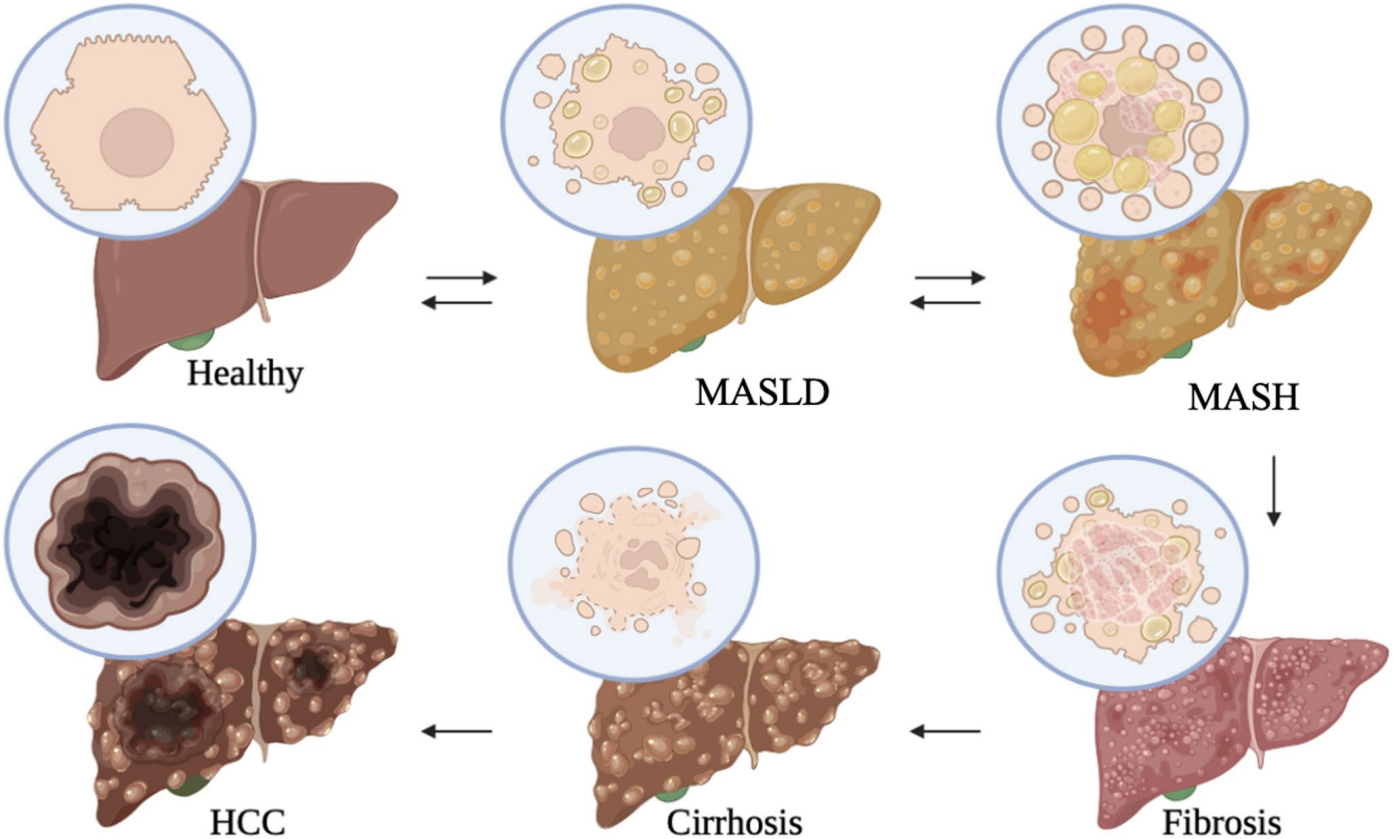
Pathogenesis and progression of SLD. Disease progression begins with excess FFA flux to the hepatocytes, causing ballooning termed MASLD; thereafter, secondary hits such as increased cytokines, endotoxins, and reactive oxygen species (ROS) cause an inflammatory response resulting in MASH. Fibrotic tissue and scarring contribute to MASH progression to liver fibrosis. Severe tissue scarring and increased necrosis characterize liver cirrhosis, which ultimately progresses to liver failure or hepatocellular carcinoma (HCC). Reprinted with permission from [40]. Copyright © 2018, Springer Nature Switzerland AG.

Liver steatosis can be triggered by various pathophysiological processes, such as impaired β-oxidation due to mitochondrial dysfunction [33, 34], elevated cytokine release, increased influx of dietary free fatty acids (FFAs) into liver cells, inadequate export of FFAs from liver cells, and *de novo* lipogenesis [35]. The pathogenesis of MASLD due to the aforementioned factors is highlighted by the interplay of several metabolic pathways [36–38]. The precise mechanisms driving the onset or progression of MASLD are still unknown [27]. MASLD encompasses a broad spectrum of fat-induced liver injuries, which range from mild SLD to cirrhosis (chronic liver damage), and eventually liver failure [39–41], as illustrated in Fig. 1.

The pathogenesis of MASLD is complex and involves the interaction of metabolic, genetic, and environmental factors that lead to fat accumulation in liver cells. In 1998, the “two-hit hypothesis” was the most corroborated theory of MASLD development [42]. This theory specifies that the initial first steps in the development of hepatic steatosis involve elevated FFAs in the portal system, increased lipogenesis, and reduced β-oxidation [43]. The “second hit” involves the increase of proinflammatory cytokines, ROS and endotoxins, which drive MASLD progression to MASH [42]. ROS induce lipid peroxidation, leading to the formation of cytotoxic products such as malondialdehyde (MDA) and 4-hydroxynonenal (4-HNE), which contribute to liver injury [44]. This damage activates key inflammatory pathways, including NF-κB and the NOD-like receptor protein 3 (NLRP3) inflammasome, thereby sustaining chronic hepatic inflammation [45, 46].

More recently, the “multiple-hit” theory has emerged as a more comprehensive explanation of MASLD development, which states that multiple etiopathogenic factors work sequentially and somewhat synergistically to promote fat accumulation in hepatocytes, leading to inflammation, fibrosis, and cellular death [47]. The activation of human hepatic stellate cells (HHSteCs) leads to an increased in the production of extracellular matrix components, particularly collagen types I and III. This excessive matrix deposition further leads to liver fibrosis, disrupting normal tissue architecture and potentially progressing MASH to cirrhosis [48, 49]. In addition to the liver, MASH affects other insulin-sensitive organs like the white adipose tissues (WATs) and muscles. These organs release adipokines and myokines, respectively, which contribute to the development and progression of MASH [50].

Concerns regarding exclusionary criteria and potentially stigmatizing terminology prompted revision of the nomenclature for NAFLD and NASH [51]. Most respondents considered the terms “nonalcoholic” and “fatty” to carry negative connotations. As a result, “SLD” was adopted as an umbrella term to reflect the diverse causes of hepatic steatosis, while preserving “steatohepatitis” as a key pathophysiological concept. This revised terminology has received broad endorsement and is intended to improve diagnostic precision and reduce stigma [51]. It is still not certain why some patients develop MASH instead of MASLD or vice versa. MASLD occurs when a patient has excess fat in the liver with little or no inflammation (simple SLD) [52]. Interestingly, MASLD does not typically progress to cause liver damage; instead, patients experience pain from the enlargement of the liver [53]. On the other hand, patients with MASH show signs of steatosis and lobular inflammation along with hepatocyte ballooning degeneration, which can be present with or without fibrosis. Often, the inflammation and liver damage of MASH leads to cirrhosis (15 − 25%), which may eventually lead to hepatocellular carcinoma (HCC) and 30 − 40% death over 10 years [54]. MASLD was reported as the most common cause of HCC among patients in 2020 [55]. This, therefore, confirms the necessity to diagnose the presence of MASLD and MASH at earlier stages and intervene with patient education and therapeutic options.

## The role of obesity in SLD and its progression

Over the past 30 years, cultural and environmental shifts in developing countries, driven by urbanization and economic growth, have led to significant changes in lifestyle. These include dietary transitions toward energy dense, processed foods and reduced physical activity, contributing to rising obesity rates [56, 57]. Increased accessibility to fast food and sedentary behaviors are key drivers of obesity and its related metabolic diseases [58].

 [58]Obesity is a worldwide epidemic with approximately 2.11 billion adults aged ≥ 25 years (45.1% of the global adult population) affected by overweight or obesity in 2021 [59]. Obesity is a major risk factor for life-threatening diseases, such as, cardiovascular diseases, hypertension, T2DM, and SLDs [60]. These diseases are primarily associated with the disruption of glucose and lipid homeostasis in several tissues, including the liver, muscle and adipose tissues, resulting in reduced insulin sensitivity [61]. The increased influx of lipids into hepatocytes has been reported to increase insulin resistance and cause hepatic steatosis. This epidemic of obesity poses a greater risk of advancing MASLD, which is present in up to 75% of overweight people, and in more than 90% of people with severe/extreme obesity [62]. This unabated increase in the prevalence of obesity and its association with MASH is a serious global threat. [63] Obesity is primarily driven by a high-calorie diet combined with a lack of exercise, leading to significant weight gain [64]. In healthy individuals, non-esterified fatty acids (NEFAs) released from dietary lipids are taken up by WAT and stored as triacylglycerols (TAGs), preventing excessive circulating NEFAs and ectopic fat deposition [65]. However, this process is dysfunctional in obesity, as hypertrophied adipocytes exhibit increased lipolysis and reduced capacity for TAG storage, causing elevated NEFA flux into the bloodstream and greater delivery of FFAs to non-adipose organs, particularly the liver. Concurrently, hyperinsulinemia and nutrient overload drive hepatic *de novo* lipogenesis while impairing β‐oxidation of FFAs, further promoting TAG accumulation within the hepatocytes. Metabolic disturbances during obesity promote the re‐esterification of excess FFAs into TAGs in non‐WAT tissues, exacerbating lipid overload and fostering hepatic steatosis [66]. Over time, persistent TAG accumulation in hepatocytes may induce cellular stress, inflammation, and progression from simple steatosis to steatohepatitis when untreated.

### Obesity-induced MASH

MASH signifies a severe condition compared to hepatic steatosis. It is marked by a pronounced accumulation of lipids in the liver, resulting in hepatocyte hypertrophy, inflammation, and increased liver scarring [67], which lead to the reduction of liver function [67, 68]. Obesity exhibits low-grade systemic inflammation, which in combination with abnormalities in fatty acid metabolism, contributes to the development of insulin resistance and dyslipidemia, which are among other key factors associated with MASH [69].

Hypertrophic WATs in the obese state produce adipokines, which mediate inflammatory, endocrine, and immune interactions in favor of insulin resistance and hepatic steatosis [70]. Previous research has demonstrated an inverse correlation between hepatic steatosis and adiponectin, an adipokine produced by adipose tissue known for its anti-inflammatory and insulin-sensitizing effects. Notably, the significant decrease in circulating adiponectin levels is noted in conditions associated with insulin resistance, such as type 2 diabetes mellitus (T2DM), MASLD and MASH [71, 72]. As hepatic steatosis advances to MASH, circulating adiponectin levels decrease further, and persists as MASH progresses to cirrhosis [72]. Resistin, a hormone involved in lipid and glucose homeostasis, is another widely studied adipokine that contributes to insulin resistance, inflammation, and MASH development [73]. Adipose tissues also produce proinflammatory cytokines such as tumor necrosis factor-alpha (TNF-α), whose levels are higher in MASH compared to hepatic steatosis, and interleukin-6 (IL-6), which promotes the development of HCC by inhibiting apoptosis and activating pro-oncogenic pathways [50].

MASH has also been described as an inflammatory subtype of MASLD. It is associated with development of dyslipidemia, T2DM, metabolic syndrome, obesity, cirrhosis [74] and the eventual need for a liver transplant [75]. The development of MASH occurs in a two-step process as described in a “two-hit hypothesis” theory, which starts with excess fat deposition in the liver because of insulin resistance or increased fat mass. This is followed by an increase in ROS-induced lipid peroxidation and inflammation in the hepatocytes [34]. This is due to the pro-oxidants generated in the mitochondria and cytochrome-P450 lipid oxidase [33, 34]. Numerous non-invasive tests and scoring systems have been developed to characterize MASH; however, liver biopsy is the gold standard for its diagnosis [76]. MASH is reversible if therapeutic intervention occurs before its progression to cirrhosis and HCC [77].

## Current therapeutics for MASLD and MASH

Lifestyle modifications, such as changes in diet and increased physical activity, with the principal goal of reducing body weight, are the centerpiece of MASH treatment [78]. Sustained weight loss shows a dose-dependent association with histological improvement in the liver, including resolution of steatohepatitis and fibrosis regression. Reviewed elsewhere, it is shown that a weight reduction between 7 and 10% improves steatosis, inflammation, and hepatocyte ballooning, while lifestyle interventions combining caloric restriction and regular moderate exercise amplify these benefits, even in the absence of significant weight loss [79]. Diet quality, particularly reduced fructose intake and adherence to a Mediterranean-style diet, as well as alcohol abstinence in patients with advanced fibrosis, are critical components of long-term management strategies aimed at slowing disease progression and improving liver-related outcomes [79]. Lifestyle modifications alone are insufficient to cause a sustainable effect. Therefore, incorporating lifestyle changes with pharmacotherapies, shown in Table 1, is often recommended [78, 80–82]. Medications prescribed for MASH treatment encompass a range of options. These include insulin sensitizers like metformin and thiazolidinediones (TZDs) such as pioglitazone, insulin secretagogues like sulfonylureas, and GLP-1 receptor agonists such as GLP-1 analogs and dipeptidyl peptidase (DPP) 4 inhibitors. Other options include inhibitors of postprandial glucose uptake in the digestive tract like α-glucosidase inhibitors such as acarbose, as well as inhibitors of renal glucose reabsorption such as sodium-glucose cotransporter-2 inhibitors like dapagliflozin [83].

Recently, the FDA approved resmetirom (rezdiffra), produced by Madrigal Pharmaceuticals (West Conshohocken, Pennsylvania, USA), as the first drug for treatment of MASH. Resmetirom activates the thyroid hormone receptor which leads to the reduction of liver fat accumulation [10]. Pegozafermin, a fibroblast growth factor 21 (FGF21) analogue, and semaglutide, a glucagon-like peptide (GLP) 1 receptor agonist, are other MASH drugs undergoing randomized clinical trials [84, 85]. These will be breakthroughs in the drug development industry and advancements for MASH patients. Previously, there were no specific treatments for MASLD and MASH. Instead, they were managed through regimens used for metabolic diseases, as they are interlinked to obesity, type 2 diabetes, and insulin resistance [86]. Lipid accumulation in the liver reduces insulin efficiency and eventually leads to insulin resistance [42]. Complications in lipid and glucose homeostasis increase the risk for cardiometabolic-related diseases [69, 86]. As a result, strategies used to treat MASH are borrowed from diseases in which adipokines and proinflammatory cytokines are involved, such as diabetes and obesity [87], and have been beneficial in reducing the consequences of SLD.

**Table 1:** Pharmacotherapies used for MASLD and MASH

Pharmacotherapy (Class)	Disease indication	Mode of Action	Side Effects	Ref
Exenatide/Liraglutide/Semaglutide (GLP-1 agonist)	Diabetes Obesity	Promotes insulin and GLP-1 biosynthesis	Hypoglycemia Pancreatitis Kidney failure	[85, 88–90]
Metformin (Biguanide)	Diabetes	Prevents glucose production in the liver	Diarrhea Hypoglycemia Lactic acidosis	[91, 92]
Pioglitazone (PPAR-γ agonist)	Diabetes	Restores insulin response by lowering blood glucose	Weight gain Bladder cancer Muscle pain	[92, 93]
Sitagliptin (DPP-4 inhibitor)	Diabetes	Promotes insulin and GLP-1 biosynthesis	Pancreatitis Heart failure Joint pain	[94]
Statins (HMG-CoA reductase inhibitor)	High cholesterol	Reduces cholesterol levels	Myositis Kidney failure Diarrhea	[95, 96]
Bupropion-naltrexone (anti-depressant and opioid inhibitor)	Obesity	Promotes increased physical activity and a reduced appetite	Nausea Diarrhea Jaundice Seizures	[97]
Orlistat (Lipase inhibitor)	Obesity	Prevents absorption of dietary fats	Bladder pain Voice loss Body aches	[98, 99]
Obeticholic acid (Farnesoid-X receptor (FXR) agonist)	Primary biliary cholangitis	Promotes improved insulin sensitivity and anti-inflammatory effects	High cholesterol Pruritus	[100, 101]
Elafibranor (PPAR-α and PPAR-δ agonist)	Diabetes	Promotes increased lipid transport and oxidation, insulin sensitivity and anti-inflammatory effects	High serum creatinine levels	[102–104]
Cenicriviroc (CCR2/5 inhibitor)	HIV/MASH	Macrophage recruitment in the liver promotes anti-fibrotic and anti-inflammatory effects	Diarrhea Fatigue Nausea	[105, 106]
Selonsertib (ASK-1/MAPK inhibitor)	MASH	Promotes anti-apoptotic, anti-fibrotic and anti-inflammatory effects	Portal pressure unimproved Nausea Sinusitis	[107]
Vitamin E (Antioxidant)	Nutrient needed for immune health	Improves hepatic steatosis, ballooning and inflammation	Adults show limited effects	[108, 109]
Resmetirom (thyroid hormone receptor agonist)	MASH	Improves hepatic steatosis, ballooning and inflammation	Diarrhea Itching Nausea Abdominal pain Dizziness	[10]

Key factors in choosing any of the above-mentioned glucose-lowering agents include their effectiveness in reducing hyperglycemia and slowing the progression of MASLD [110]. Peroxisome proliferator-activated receptor (PPAR) agonists increase circulating adiponectin levels and improve liver histology in patients living with MASH [82]. One-year treatment with metformin has been shown to lower alanine aminotransferase (ALT) and aspartate aminotransferase (AST) levels while increasing serum adiponectin levels in MASLD patients [5]. TZDs such as pioglitazone and rosiglitazone, have also demonstrated improvements in adiponectin levels [111]. TZDs have the strongest evidence for efficacy in treating MASLD/MASH, however, concerns about long-term cardiovascular risks and other side effects limit their approval for MASLD treatment [111]. Additionally, cholesterol-lowering drugs, like statins, are being explored as potential therapies for MASLD due to their ability to inhibit 3-hydroxy-3-methylglutaryl-CoA (HMG-CoA) reductase [41]. Statins increase adiponectin levels and manage MASLD by regulating dyslipidemia [112]. While insulin-sensitizing agents and lipid-lowering medications show promise for liver health and type 2 diabetes, they are typically used for other indications beyond MASLD [63].

Novel drug classes are also being tested in clinical trials for MASH, including selonsertib, cenicriviroc, semaglutide and elafibranor [85, 90, 106, 107]. Selonsertib, a selective inhibitor of apoptosis signal-regulating kinase 1 (ASK1), exhibits anti-inflammatory and antifibrotic properties, showing promise in reducing fibrosis in MASH and stage 2–3 fibrosis [113]. Analogously, cenicriviroc, an antagonist of the C-C motif chemokine receptor 2/5, is involved in macrophage recruitment in the liver and also demonstrated anti-inflammatory and antifibrotic effects in MASH patients with fibrosis [114, 115]. Semaglutide showed anti-MASH and antifibrotic without worsening fibrosis potential in phase 3 clinical trial. The drug also resulted in greater weight loss but was associated with gastrointestinal side effects [85]. Elafibranor, a dual agonist of PPAR-α/δ, was reported to resolve MASH and improve hepatic steatosis, ballooning, lobular inflammation, and fibrosis in MASLD patients with a fibrosis score ≥ 4. This drug also demonstrated broad spectrum activity by improving the lipid profile, markers of inflammation, and ameliorated insulin sensitivity [104]. Due to shortfalls in current therapeutic interventions and the fact that only one drug is approved for MASLD/MASH, targeted therapy is emerging as an probable solution for their treatment.

### Targeted therapy for MASLD

Targeted therapy has revolutionized disease treatment, which is especially evident in current cancer treatment strategies. It relies on the use of molecules that specifically target biomarkers that are exclusively or highly expressed in diseased cells/tissues compared to healthy ones [116]. Biomarkers are involved in various stages of disease development and progression; thus, interference with their actions can reverse the effects of the disease [11, 116]. Monoclonal antibodies, peptides [11], and aptamers are among the targeting moieties that have been explored in targeted therapy. Similarly, studies have highlighted the importance of using targeted therapies for various metabolic diseases, such as obesity and type 2 diabetes [11, 13, 117].

Drug targeting and delivery can be categorized into passive and active targeting. Passive targeting is a common approach in cancer treatment that delivers drugs to tumors by taking advantage of their leaky vasculature, allowing the drugs to accumulate in the tumor microenvironment [118]. On the other hand, active targeting refers to specific interactions between the drug and the target cells via specific ligand‒receptor interactions [118]. In this strategy, drugs are linked to targeting molecules like antibodies, aptamers, or peptides to achieve more precise and effective therapeutic strategies [119]. The specific delivery offered by targeted therapies allows patients to experience reduced drug dosage as well as side effects. Targeted therapy is a promising treatment approach that is practical and continues to be explored to combat chronic and infectious diseases [118, 120, 121]. The potential application of targeted therapy in MASLD and MASH is therefore motivated by the advances made in obesity treatment.

#### Targeted therapy for obesity

The relationship between cancer and obesity remains unclear, yet the similarities in their development and progression are the basis of their similar treatment strategies [122], and continue to influence the development of novel drug leads for obesity. Like cancer, obesity development follows an uncontrollable growth (increase in size and number) of adipocytes in the WATs, to an extent that obesity is regarded as an endocrine tumor. Therefore, it makes sense why treatment strategies for cancer would work for obesity [123] and vice versa, where obesity treatments are now also investigated for cancer treatment. In a recent study, for instance, activation of brown adipose tissue in mouse xenografts reduced tumor growth and glucose uptake in a patient with Hodgkin’s lymphoma [124].

**Fig. 2:**
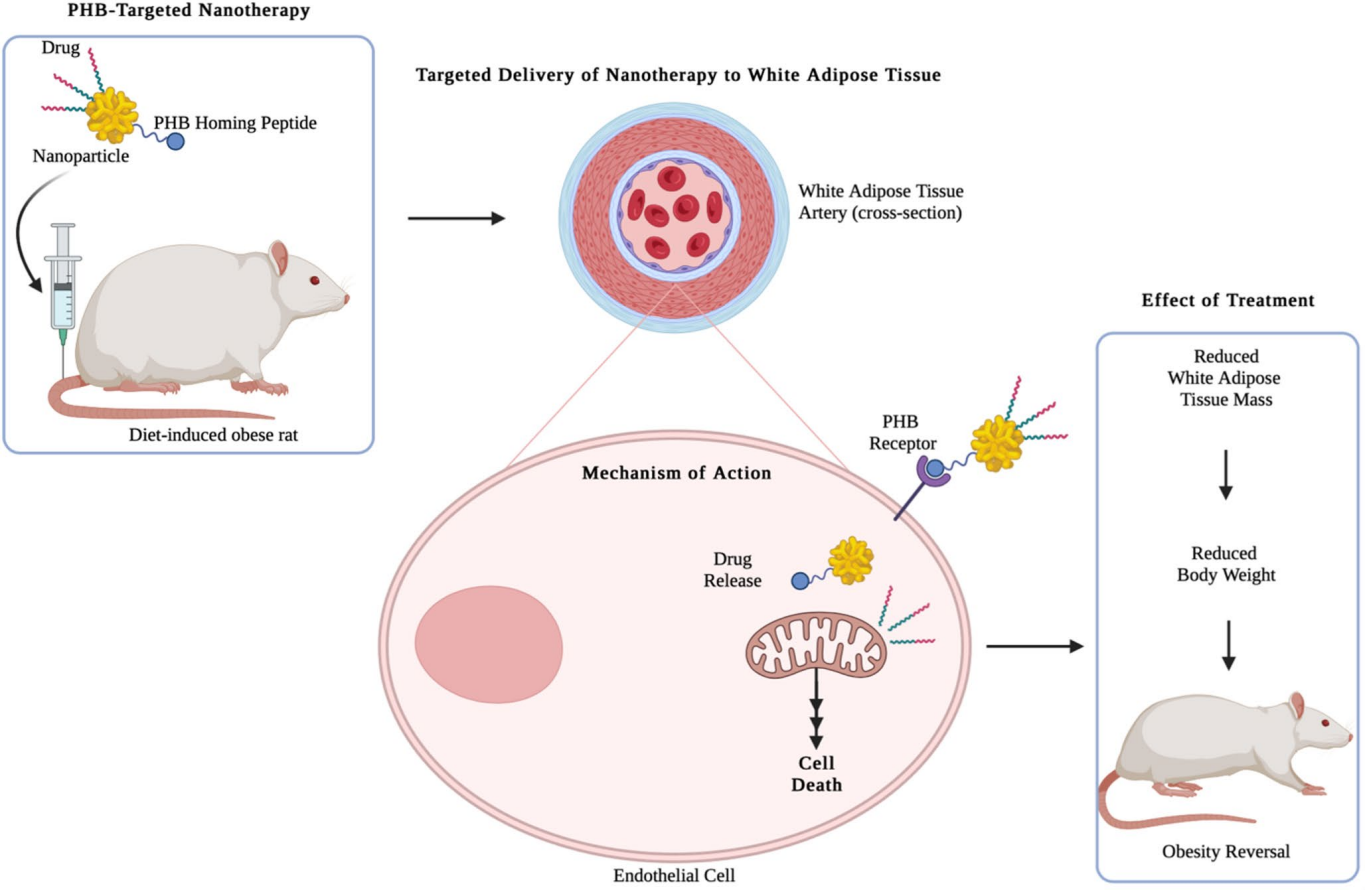
PHB-targeted nanotherapy for reversing obesity in diet-induced rats involves the use of PHB-targeted nanoparticles that bind to the PHB receptor on cell surfaces. Once bound, these nanoparticles are internalized into the cytoplasm of PHB-expressing endothelial cells. This internalization results in the release of the therapeutic drug, triggering cytochrome C release from the mitochondria, which then activates caspases and induces apoptosis. This apoptotic process leads to the reversal of obesity. Reprinted with permission from [131]. Copyright © 2019, Springer Nature Switzerland AG.

Targeted therapy has shown tremendous success in cancer and is now also explored for the treatment of obesity with exciting outcomes. PHB, a protein highly expressed in the vasculature of obese mice [125] and rats [126], is the most experimentally investigated biomarker for obesity. Accumulation of fat in adipose tissues increases adipogenesis, which is always coupled with increased angiogenesis for sustenance in growth and expansion [127, 128]. Thus, inhibiting angiogenesis in these highly vascularized WATs by cutting down the blood supply will starve the adipocytes and force them to metabolize the stored fat, reduce WAT weight and reverse obesity and its related comorbidities, as illustrated in Fig. 2 [128]. When a ligand (adipose homing peptide - CKGGRAKDC, AHP) that targets PHB was attached to biomaterials, it accumulated in PHB-expressing cells [128] and tissues [129]. This was also reported in diet-induced obese mice, where AHP attached to a proapoptotic peptide (KLAKLAK) reduced WAT weight and body weight [125]. Targeting the WAT vasculature also improved the metabolic activities in the mice, i.e., glucose tolerance and insulin sensitivity [125]. This was consistent with the health benefits associated with anti-obesity drugs, with 5–10% weight loss being sufficient to restore some of the metabolic activities. While anti-obesity drugs are effective in reducing body weight, they are limited by non-specificity that can be overcome by targeted therapies. Further improvements in the targeted strategies were achieved by using drug delivery systems to protect the drugs from biodegradation, early drug clearance, and eliminate toxic effects on normal cells and tissues [121, 130]. Effective drug delivery systems must align with four key requirements: (1) loading sufficient amount of drug of interest, (2) adequate blood circulation and residence time to reach the target site, (3) drug retention and (4) drug release at the desired site. Conventional drug delivery systems have shown improved drug efficacy by delivery of drugs at the desired rate for extended periods [121].

**Fig. 3:**
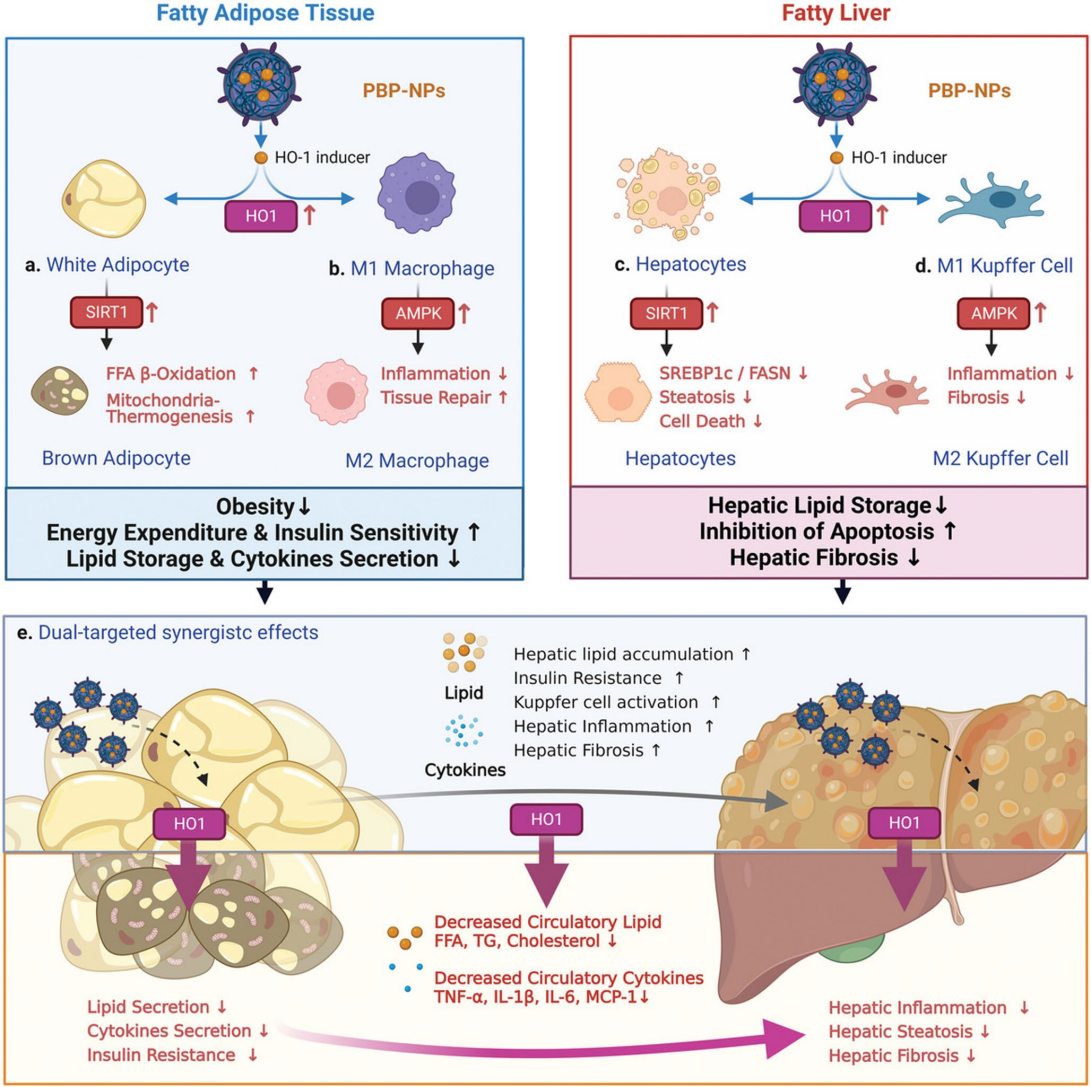
Dual-targeted effects of AHP-PLGA-hemin/ CoPP in obesity-induced T2DM and diet-induced NASH mouse models. AHP-PLGA-hemin/ CoPP reversed the metabolic dysfunction by **(a)** conversion of WAT into BAT via the SIRT1 pathway, **(b)** switched M1 macrophages into anti-inflammatory M2 macrophages via AMPK signaling, **(c)** reduce expression of FASN and SREBP1c, **(d)** reduce inflammation by switching M1 Kupffer cells into M2 Kupffer cells and activate hepatic stellate cells, **(e)** reduction of systemic lipids and cytokines from WAT, hepatic steatosis, inflammation, and fibrosis. Reprinted with permission from [21]. Copyright © 2022, Wiley.

Nanomaterials are among the drug delivery systems previously reported to be effective for targeted drug delivery in obesity to enhance anti-obesity drug effects [132]. PHB-targeted drug delivery and localization in the WAT vasculature was achieved using various types of nanomaterials, such as gold nanoparticles (AuNPs), quantum dots (QDs), liposomes, and polymeric NPs [128, 133, 134]. In addition to drug delivery, metallic NPs offer additional benefits due to their unique physicochemical properties. Their optical properties allowed them to also be used as photosensitizing (AuNPs) [135] and as imaging (QDs) [129] agents. All these strategies are summarized elsewhere [131], and only the benefits of using nanocarriers in targeted therapy are discussed in this review. Among others, NPs can passively target diseased cells due to the enhanced permeability and retention (EPR) effect, transport insoluble drugs, and be monitored in real time. Moreover, NPs have a large surface area and can carry multiple biomolecules. To increase their selectivity, targeting moieties such as antibodies and aptamers are often attached to their surface [136]. Nanomaterials were targeted to PHB [137] and adipocyte plasma membrane-associated protein [138] using AHP and adipo-8 aptamers, respectively, resulting in significant weight loss. These nanomaterials were efficiently delivered to the target and protected the anti-obesity drugs from premature adsorption or systemic clearance. Their ability to reach metabolic tissues [14] and reduce ectopic fat deposition in the liver [139] also suggests that these strategies will also be effective for MASLD and MASH as demonstrated in Fig. 3. In the study, poly(lactic-co-glycolic acid) (PLGA) NPs were loaded with heme oxygenase-1 (HO-1) inducers (iron protoporphyrin ix (hemin) or cobalt protoporphyrin ix (CoPP)) and surface modified with AHP then used for the treatment of obesity-induced T2DM and diet-induced MASH in mice models. AHP-PLGA-hemin/ CoPP targeted the same biomarker (PHB) in the two disease models and reversed the disease effects [21].

#### Targeted therapy for MASLD and MASH

The link between MASLD/MASH and other metabolic diseases, especially obesity and type 2 diabetes, suggests that targeted treatment strategies employed against these diseases may also lead to an improvement in hepatic ailments [86]. In fact, MASLD/MASH uses treatment regimens for these diseases (Table 1), focusing on the reduction of WAT mass and promotion of insulin sensitivity. PPAR agonists (elafibranor and lanifibranor) and semaglutide reached phase III clinical trials for the clinical treatment of MASLD and were reported to have a positive response in hepatic steatosis, MASH and fibrosis [49, 85, 140]. Although these strategies can reduce the deleterious effects of SLD, they unfortunately lack specificity, harbor adverse side effects, and lack efficacy [140]. SLD demands robust and disease-specific strategies that will be able to treat this multifaceted disease. These attributes support that targeted therapy can be an alternative therapy and provide sustainable effects in the fight against MASLD and MASH.

Efforts to study the molecular mechanisms of MASLD and MASH identified some anatomical disease features, hepatic stellate cells were mainly affected when compared to other liver components, such as hepatocytes, Kupffer cells and hepatic sinusoidal endothelial cells [141]. Specifically, PPAR is highly expressed by stellate cells and FXR in the hepatocytes. In addition, sterol regulatory element-binding protein 1c (SREBP-1c), apolipoprotein C3, adenosine monophosphate-activated protein kinase (AMPK) and nuclear factor erythroid 2-related factor 2 (Nrf2) are highly expressed during injury to the liver by MASLD/MASH [86, 140]. It is therefore not surprising why treatments that target these molecules were also effective in the management of MASLD/MASH.

Protease-activated receptor-2 (PAR2) is involved in a signaling pathway of liver inflammation and fibrosis, and targeting this receptor was able to reverse the disease effects. In a diet-induced MASLD mouse model, injection of PAR2 pepducin into the mice inhibited progression of hepatic fibrosis. This intervention was associated with reduced fat deposition, inflammation and fibrosis in the liver [141]. In search of MASLD-specific biomarkers, NAFLD01 aptamers were selected using Systematic Evolution of Ligands by EXponential. These aptamers bound to the cell membrane in an in vitro model of MASLD which was established using human hepatocellular carcinoma (HepG2) cells [142]. The aptamers were later demonstrated to target CD36 receptors in MASLD models (HepG2, mouse and human liver tissues). The CD36-specific aptamers showed dual activities and could target and improve lipid deposition in HepG2 cells. Thus, CD36 receptors could serve as a potential target for MASLD-targeted therapeutic intervention [143].

**Fig. 4:**
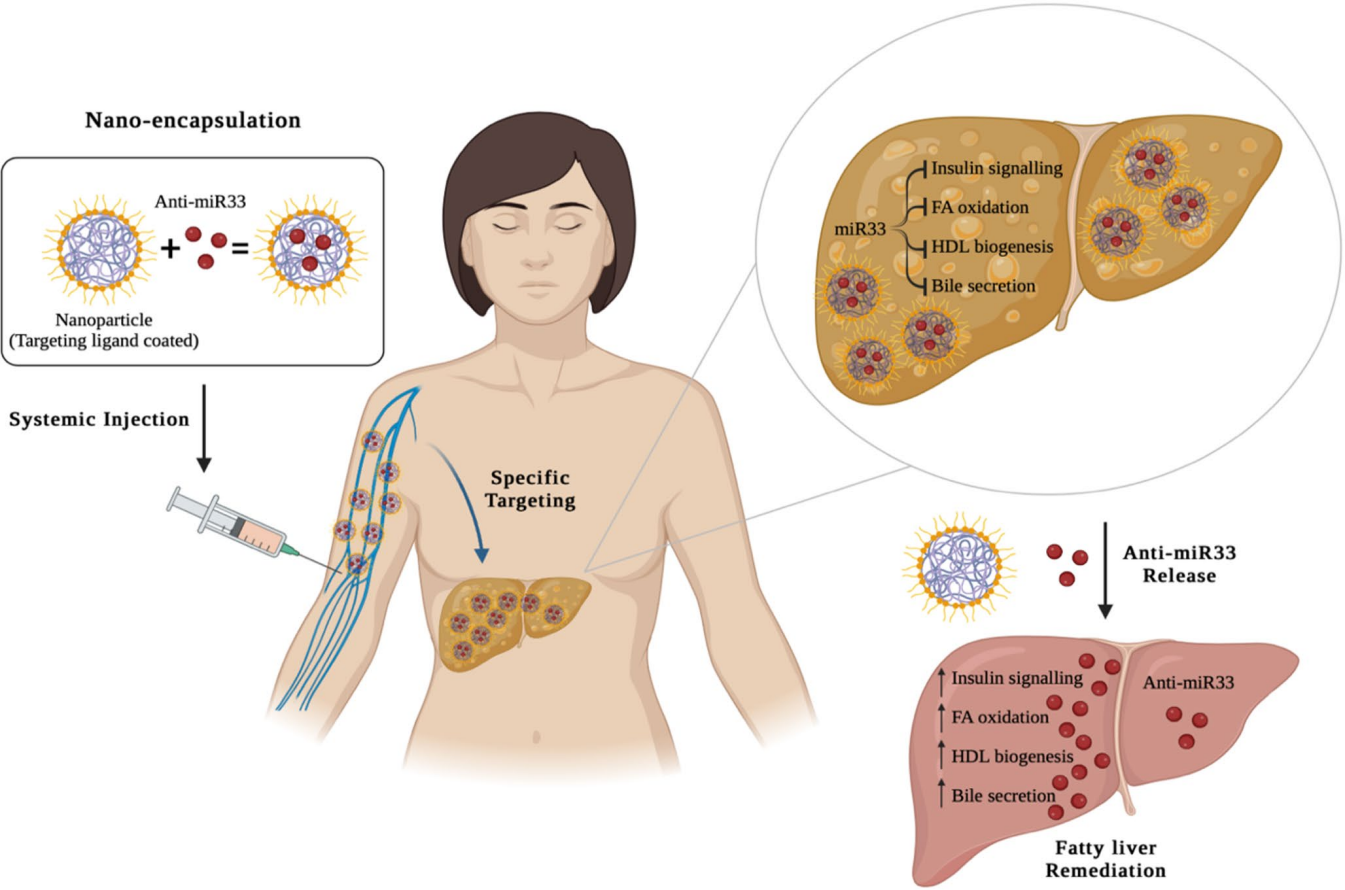
Nanobased targeted delivery of anti-miR-33 to hepatic cells. Increased miR-33 levels inhibited the activation of genes that promote insulin signaling, fatty acid oxidation, high-density lipoprotein production, and bile secretion in the liver. Consequently, the introduction of anti-miR-33 effectively counteracts these unfavorable metabolic effects of miR-33 and addresses fatty liver concerns. Systemic injections of nanoparticle-encapsulated anti-miR33 successfully alleviated fatty liver conditions. Liver targeting ligands were attached to the nanoparticles to ensure precise targeting of the liver. Reprinted with permission from [144]. Copyright © 2019, Elsevier Ltd.

MicroRNAs (miRNAs), a class of small noncoding RNAs, also showed potential as therapeutic targets for MASLD and MASH by targeting pathways involved in cellular proliferation and differentiation, insulin secretion, and lipid and energy metabolism. The miRNAs involved in various stages of MASLD/MASH pathogenesis and progression, together with their possible targets are reviewed in the literature [144]. Studies have demonstrated that by using antisense oligonucleotides (anti-miRs), it is possible to target specific miRNA transcripts and disrupt their biological functions [145–148]. miR-33 plays various roles in metabolic syndrome (atherosclerosis, obesity, liver fibrosis, insulin resistance) by regulating fatty acid oxidation, insulin signaling and glucose metabolism pathways. Targeting miR-33 with anti-miR33 reversed adverse metabolic effects related to MASLD and obesity [144]. The challenges associated with miRNA-based therapy have been highlighted together with strategies to counteract them [144]. Nanocarriers are among the strategies that can enhance stability and deliver miRNA at the target site [145]. This was further proposed for systemic delivery of anti-miR-33 to the liver, where liposomes could be used as a drug delivery system. Nanoencapsulation of anti-miR-33 might increase drug delivery at the target site, and influence genes related to miR-33 functions such as insulin signaling, fatty acid oxidation, high-density lipoprotein biogenesis and bile secretion, subsequently reversing SLD, as shown in Fig. 4 [144].

Nanocarriers have many advantages over other drug delivery due to their small size (1–100 nm) and their larger surface area. They are classified as organic and inorganic nanomaterials; all are capable of loading multiple molecules. Additionally, they are small enough to transverse through cellular barriers with and without the aid of targeting moieties [145]. Studies illustrating the beneficial effects of various nanocarriers for in vitro and in vivo models of metabolic diseases are summarized in Table 2, Anti-obesity PHB-targeted strategies not only reduced fat content in 3T3-differentiated adipocytes and diet-induced obese mice but also ameliorated hepatic inflammation and toxicity [20, 149]. AHP-PLGA-hemin/ CoPP enhanced expression of HO-1 resulting in reduced body weight that was accompanied by improved metabolic activities (reduced blood glucose) in obese mice that were treated once a week for four weeks. These effects lasted for seven weeks after cessation of treatment. The same treatment reduced inflammation, steatosis and fibrosis in MASH model [21].

**Table 2:** Nanocarriers in the treatment of metabolic diseases

Disease	Targeting moiety	NP type	Therapeutic molecule	Mechanism	Outcome	Ref
Obesity	AHP	Liposomes	Triiodothyronine (T3)	Conversion of WAT to BAT	Increased expression of WAT browning markers (uncoupling protein 1 (Ucp1) Reduced AT macrophage infiltration and expression of proinflammatory cytokines Prevent cardio- and hapato-toxicity of T3	[149]
Obesity	AHP coupled with octaarginine	Liposomes	Rosiglitazone	Conversion of WAT to BAT, consequently increasing energy expenditure and reduction of AT weight	Increased expression of WAT browning markers (Ucp1 and cytochrome c oxidase polypeptide 7a1 (Cox7a1)) in vitro Reduce body weight and hepatic toxicity in DIO mice Reduce inflammation by Switching of adipose tissue-derived macrophages phenotype into anti-inflammatory M2 Types	[20]
Obesity T2DM MASH	AHP	PLGA	Hemin CoPP	Stimulation of HO-1 activity	Conversion of WAT to BAT Increase FFA β-oxidation and insulin sensitivity Reduce liver inflammation, steatosis and fibrosis	[21]
MASLD/ MASH	Passive targeting	Drugs co-assembled into nanofibers	Ketoprofen Fenofibrate	Inhibition of cyclooxygenase-2 (ketoprofen) and activation of PPAR-α (fenofibrate)	Reduced in vitro and in vivo lipid accumulation and triglyceride Reduced liver ballooning and inflammation	[150]
MASLD/ MASH	Passive targeting	PLGA	Rapamycin	Reduction of SREBP-1c-mediated *de novo* lipogenesis and activation of PPARα-dependent fatty acid oxidation	Upregulate expression of PPAR-α, Cpt-1α, and Pgc-1α	[151]

In a study by Wang and colleagues, it was demonstrated that nanomaterials can enhance drug bioavailability, targeting and efficacy. The fenofibrate and ketoprofen nanofibers reduced lipid accumulation and triglyceride content in HepG2 cells; in vivo, the nanofibers passively targeted the liver and reduced inflammation and liver injury associated with MASLD and MASH. The nanofibers greatly enhanced the bioavailability of poorly soluble fenofibrate and reduced hepatic steatosis and its associated inflammatory response [150]. Loading of rapamycin on polymeric NPs improved drug uptake by HepG2 cells, resulting in a reduction in lipid accumulation stimulated by PPAR-mediated fatty acid oxidation and a reduction in SREBP-1c-dependent *de novo* lipogenesis [151]. Some of the liver targets that are implicated in various stages of MASLD/MASH and their ligands are summarized in Table 3, as reviewed elsewhere [11, 152]. However, more research is warranted, as most if not all these targets are not organ-specific and are expressed in multiple locations. The success of targeted strategies relies on their ability to target and deliver drugs to specific locations and impose their bioactivity on desired targets.

**Table 3:** Biomarkers that are implicated in development and progression of MASLD/MASH

Target liver component	Target	Targeting ligand	Ref
Hepatocytes	Asialoglycoprotein Receptor	GalNAc	[153]
		galactose, lactose and glucose	[154]
	FXR	Farnesol Bile acids (chenodeoxycholic acid, deoxycholic acid, lithocholic acid, cholic acid and obeticholic acid) Cilofexor Tropifexor	[155]
	CD36	NAFLD01 aptamers	[143]
	Organic Anion Transporting Polypeptides	Estrone-3-Sulphate	[156]
	Heparan sulfate proteoglycans	Conserved region I from Plasmodium sporozoites circumsporozoite protein	[157]
	PPARα	Fenofibrate Clofibrate	[158]
	Transferrin receptor	Transferrin	[154]
Kupffer cells	Mannose receptor C type 1	Mannosyl units in albumin	[154]
	Gprotein coupled bile acid receptor (Gpbar1 or TGR5)	Deoxycholic acid Lithocholic acid	[159]
Hepatic stellate cells	CD44	Hyaluronic acid Chondroitin sulfate	[154]
	Integrin ανβ3	cRGDyK	[154]
Liver sinusoidal endothelial cells	Intercellular adhesion molecule-1 (ICAM-1) Intercellular adhesion molecule-2 (ICAM-2) Vascular cell-adhesion molecule (VCAM) -1		[160]
	Mannose receptor	Mannan	[154]

## Future perspectives

Targeted therapy holds promising prospects for MASLD and MASH, and their molecular pathways have led to the identification of biomarkers that can be used as targets for therapeutic interventions [18– [21, 161]. As we delve deeper into the understanding of the molecular intricacies underlying the disease and progress made thus far, it becomes evident that this strategy could be key to effective treatment. To attest to this, there were some advances made in using targeting molecules (antibodies, peptides, aptamers, etc.) that selectively target factors that are involved in the development and progression of the disease, including pro-inflammatory cytokines, fibrogenic markers, or miRNAs [17, 144], as described earlier. Among those clinically tested is the asialoglycoprotein receptor, where N-acetylgalactosamine (GalNAc) was used to transport revusiran to the liver of healthy volunteers. Revusiran is a small interfering RNA that inhibits expression of transthyretin (TTR). Thus, GalNAc-revusiran conjugate reduced TTR systemic expression and confirmed delivery of the conjugate into the hepatocytes [153].

Targeting offers a versatile platform for achieving precision medicine. While all the targeting molecules are efficient, some have some limitations that can reduce their therapeutic efficacy, such as specificity and biodegradation. Aptamers, which are single-stranded nucleic acids or peptides that can selectively bind to specific molecular targets, are highly recommended. Their unique properties, such as high binding affinity, low immunogenicity, and ease of modification, make them ideal candidates for the development of highly efficient targeted therapies [162–164]. Aptamers can have multiple functions as both targeting, drug delivery and therapeutic agents [12, 163, 164]; in this regard, aptamer-based therapies have the potential to not only stop the progression of steatohepatitis but also reverse the condition. Furthermore, the bioactivities of aptamers can be complemented by other technologies, to produce systems with enhanced drug targeting and sustainable effects. Limitations of the drugs that have beneficial health effects in MASH (Table 1) can be reduced through drug delivery systems to target the diseased tissues. Overall, the future of steatohepatitis treatment is intimately intertwined with innovative technologies (aptamers and nanotechnology) that can offer new avenues for personalized interventions for this challenging liver disease.

## Conclusion

The liver is responsible for several bodily functions, including detoxifying blood in circulation. However, in an obese state, liver function is impaired through the influx and accumulation of FFAs in the hepatic tissue. Although there have been great advances in finding treatments for SLDs, many of these have fallen short in that they lack efficacy and do not act specifically on the liver. Targeted treatment strategies have been employed in recent years and have shown great promise in disease prevention and treatment. Inflammation and oxidative stress are key mediators of diet-induced obesity and MASLD/MASH; thus, identifying biomolecules involved in these processes may serve as potential biomarkers for targeted therapy. This review has highlighted the impact of targeted therapy on obesity and the possibility of using this therapeutic strategy in SLDs, especially MASLD/MASH. MASLD/MASH is a multifactorial disease involving multiple pathways and molecular targets that also play part in several other diseases; hence, the current therapeutic targets are involved in several metabolic diseases. Targeting and modulation of multiple disease-related molecules might offer synergistic and comprehensive therapeutic approaches for various ailments. This has been a challenge for drug development, as most diseases are regulated through similar pathways. By targeting multiple disease-related molecules, treatment specificity might be difficult to control. Strategies targeted at fibrosis, lipid metabolism and inflammatory pathways are successful in improving MASLD/MASH; hence, more specific hepatic targets for these pathways require further exploration. Targeted therapy can provide hope to end the rise in SLDs.

## Data Availability

No datasets were generated or analysed during the current study.
